# SEM and AFM Studies of Two-Phase Magnetic Alkali Borosilicate Glasses

**DOI:** 10.1155/2017/9078152

**Published:** 2017-03-27

**Authors:** N. Andreeva, M. Tomkovich, A. Naberezhnov, B. Nacke, A. Filimonov, O. Alekseeva, P. Vanina, V. Nizhankovskii

**Affiliations:** ^1^Peter the Great Saint-Petersburg Polytechnic University, Polytechnicheskaya 29, St. Petersburg 195251, Russia; ^2^Ioffe Institute, Polytechnicheskaya 26, St. Petersburg 194021, Russia; ^3^Leibniz University of Hannover, ETP, Wilhelm-Busch-Street, 30167 Hannover, Germany; ^4^International Laboratory of High Magnetic Fields and Low Temperatures, Gajowicka 95, 53-421 Wroclaw, Poland

## Abstract

The morphology and composition of four types of two-phase alkali borosilicate glasses with magnetic atoms prepared by inductive melting have been studied. The results of scanning electron microscopy point to uniform distribution of Na, Si, and O atoms in these samples while magnetic iron atoms form ball-shaped agglomerates. The magnetic properties of these agglomerates have been confirmed by magnetic force microscopy. Atomic force microscopy had shown that in these samples two different morphological structures, drop-like and dendrite net, are formed. The formation of dendrite-like structure is a necessary condition for production of porous magnetic glasses. The obtained results allow us to optimize the melting and heat treatment processes leading to production of porous alkali borosilicate glasses with magnetic properties. The first results for nanocomposite materials on the basis of magnetic glasses containing the embedded ferroelectrics KH_2_PO_4_ demonstrate the effect of applied magnetic field on the ferroelectric phase transition.

## 1. Introduction

Mesoporous silica materials, especially alkali borosilicate glasses with magnetic properties, attract undiminishing interest because of the magnificent physicochemical properties such as a low thermal expansion coefficient, high chemical and mechanical resistance, a large and controllable porosity, a high surface area, tunable pore sizes and volumes, and optical and magnetodielectric characteristics [1–12]. These glasses have silanol groups on the pore surface for modification, which provides a robust framework (matrix) for deposition and incorporation of guest molecules to produce multifunctional materials with unique capabilities and properties [13]. For example, they can be used for laboratory equipment and glass cookware, magnetooptical switches, and drug delivery with controlled release and bioseparation [14–21], hyperthermia application [22], bone tissue regeneration [23], and other medical applications [24–26]. The immobilization of proteins on the porous silica opens a great potential for applications of these materials in biology as biosensors and for biocatalysis [27–29]. In addition these matrices can be used as a model object for creation of new multifunctional materials with very developed interface, for example, multiferroics [7], by filling of pores and we can regulate the average size of embedded nanoparticles in a nanometer scale [7, 11, 27]. One of the first attempts to create porous alkali borosilicate glasses with magnetic properties has been undertaken in the papers [4, 5]. It has been shown that there is an optimal concentration of a base mixture at which an initial hematite (*α*-Fe_2_O_3_) transforms thoroughly into magnetite (Fe_3_O_4_) at melting in a platinum crucible. It should be noted that modern methods of etching of chemically unstable phase permit producing the porous glasses with developed interconnected dendrite pore net and controllable average pore diameters. Skeleton of porous glasses consists of amorphous silica that is an additional advantage of this material. This fact simplifies significantly analysis of temperature evolution of crystal structure of embedded materials by X-rays and neutron diffraction. Unfortunately, the conventional methods of magnetic porous glass production are sufficiently expensive and do not permit obtaining a large amount of these glasses per one melting circle. We have developed new approach to production of similar magnetic glasses, the induction melting [29]. Our measurements confirmed magnetic properties of these two-phase (nonporous) glasses [29], but an existence of dendrite structure formed by chemically unstable phase was under question. It should be noted that this structure is necessary condition for formation of multiplying connected channel (pores) system at chemical etching. So we can formulate the following principle questions of these study:

- Does a dendrite structure exist in our glasses?
- Could we achieve the uniform agitation of components at induction melting?
- What is the kind of melting process parameters and heat treatment regimes that we have to use for production of the two-phase glasses with optimal combination of magnetic properties and dendrite structure?
- Is it possible to govern by ferroelectric phase transition in nanocomposites on the basis of these glasses using external magnetic field?

In our measurements we have used atomic force microscopy (AFM) including magnetic force microscopy (MFM), scanning electron microscopy (SEM), and dielectric spectroscopy (including at magnetic fields up to 10 T) as experimental methods.

## 2. Materials and Methods

Using an initial mixture 60% SiO_2_: 15% B_2_O_3_: 5% Na_2_O: 20% Fe_2_O_3_ (pointed out the molar percentages) we have prepared four types of ferriferous alkali borosilicate glasses. Melting was carried out at different temperatures in the interval 1450–1550°C following to the procedure described in the paper [29]. The sample number 1 (S1) was cooled at the rate 5°C/min from the melted state down to room temperature, the samples numbers 2–4 (S2, S3, S4) were cooled with the same rate down to 560°C and were withstood at this temperature during *T*_ph_ = 120 hours for phase separation. The principle difference between these samples (Table 1) was in melting temperature (*T*_melt_) and duration (*T*_dur_) of this process because one of the purposes of this study was the optimization of melting process. In every case we have produced an ingot of glass with diameter ~60 mm and height ~70 mm. Thereupon from the central part of ingot we have cut out the thin plates with thickness ~2 mm after that the sample surfaces were polished. Structure and composition of samples have been studied by electron scanning microscopy Quanta 200 with X-rays microanalyzer EDAX; the surface morphology and distribution of magnetic properties over the sample surface were studied with atomic force microscope MFP 3D (Asylum Research, CA). Measurements by MFM were done in external magnetic field applied in the sample plane in the range of ±0.75 T. Unfortunately there was no possibility of applying the magnetic field perpendicular to the sample plane and above 0.75 T. The temperature dependence of dielectric permittivity of nanocomposites on the basis of macroporous S3 glasses embedded into the pores ferroelectrics KH_2_PO_4_ had been carried out at 1 kHz at zero and* B* = 10 T magnetic fields.

## 3. Results and Discussion

**Figure 1:**
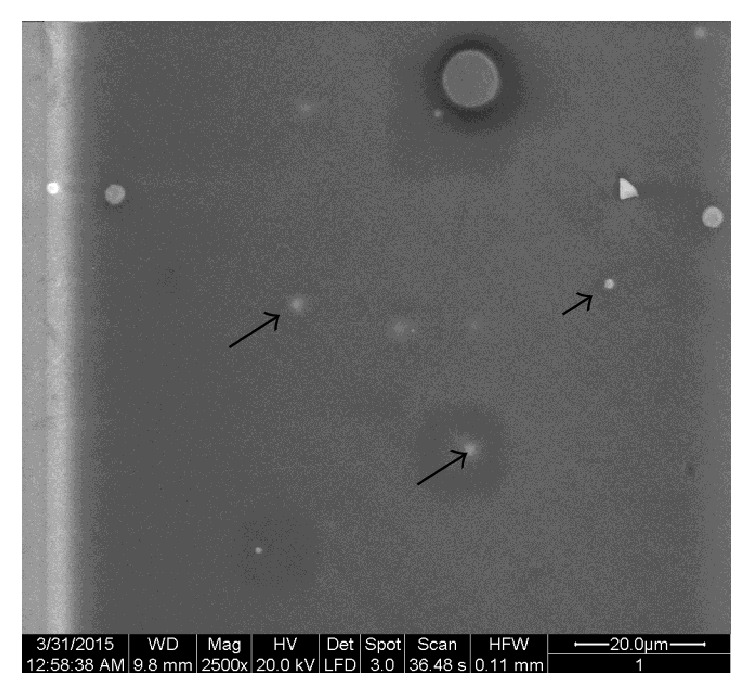
SEM image for S1 glass.

**Figure 2:**
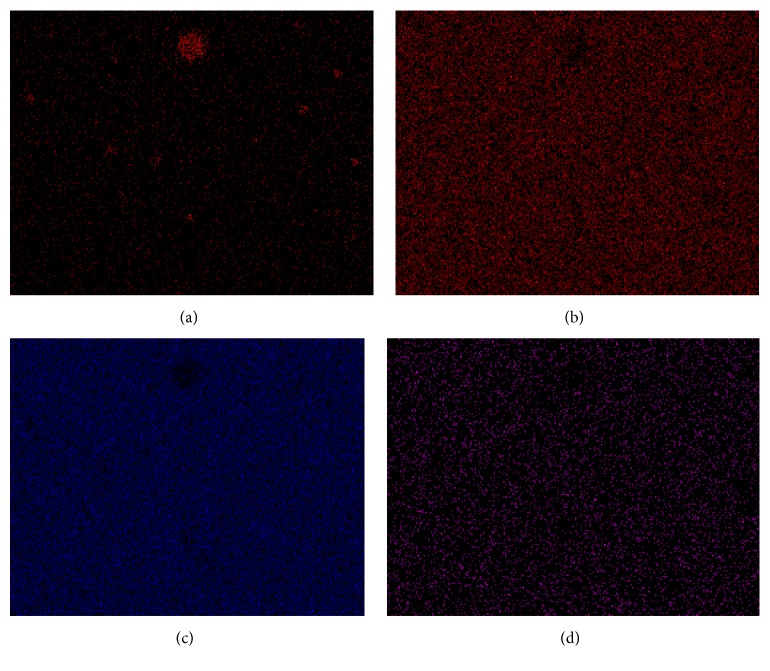
Element distribution in Figure 1: (a) Fe, (b) O, (c) Si, and (d) Na. The scale is shown in Figure 1.

SEM image in Figure 1 is typical for all our samples and it is easy to see the large quasi-spherical inclusions (agglomerates) with different diameters, but the most parts of these agglomerates have the characteristic diameters of 1 *μ*m (black arrows). Element analysis has shown that Fe concentration in these inclusions is very high, but outside of agglomerates the iron atoms are distributed uniformly (Figure 2(a)). We have already observed in [4] an appearance of large iron-containing agglomerates in two-phase magnetic glasses, produced by conventional melting in a platinum crucible with mechanical agitation. As it has been shown in [4, 5] the iron-containing inclusions are a result of self-assembling of Fe_3_O_4_ monodomain nanoparticles with average size of 16 ± 2 nm. Moreover, we have shown [4] that considerable part of iron concentrating in the so-called chemically unstable phase (CUP) is removed at etching and forms the randomly oriented dendrite net of channels. Hence, the uniform distribution of iron atoms outside of agglomerates was expected as one can see in Figure 2(a). The similarity of glasses produced by conventional method and by an induction melting is confirmed also by homogeneous distribution of O, Si, and Na (Figures 2(b)–2(d)) on the sample surfaces. The element concentrations in our samples (from analysis of X-rays data) are presented in Table 2.

Appearance of carbon associates with using of graphite crucible because at melting carbon of crucible dissolves partly in the melt. Generally, this admixture is undesirable and in future we are going to use high-temperature ceramic crucibles, but this time the carbon atoms play a role of independent markers, indicating the uniform agitation of melt due to convection and application of high frequency electromagnetic field.

**Figure 3:**
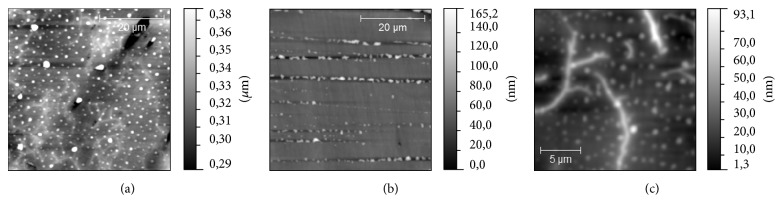
Topography of glasses: (a) S2, (b) S3, and (c) S4.

**Figure 4:**
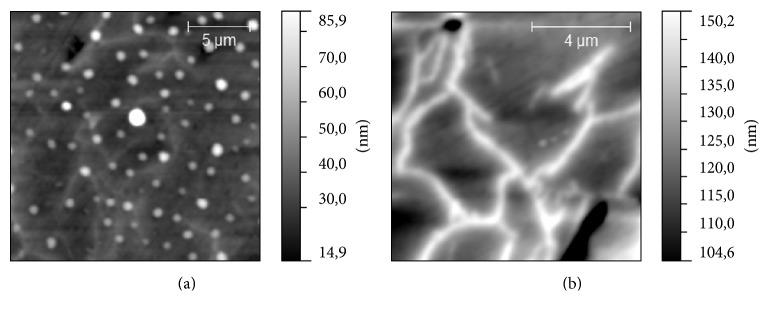
Topography of S2 sample: (a) drop-like surface structure; (b) dendrite-like surface structure.

**Figure 5:**
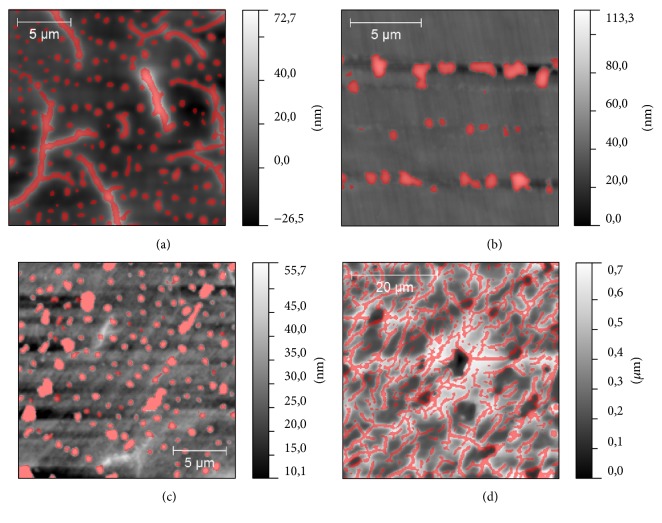
Surface topography of samples with dendrite-like and drop-like structures marked with color mask. (a) S4 sample: the mixed structure occupied from 15 to 20% of the surface; (b) S1 sample: jagged dots are concentrated in the scratches and occupied from 5 to 10% of surface; (c) drop-like structure of S2 sample: it occupied from 5 to 15% of surface; (d) dendrite-like structure of S2 sample: it occupied up to 30% of the surface.

**Figure 6:**
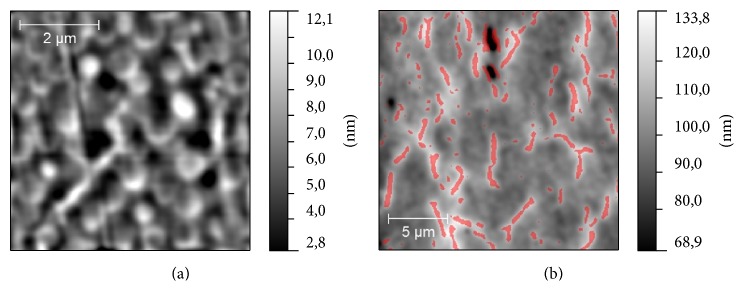
Topography of S3 sample on a different scale: (a) grain-like structure is shown; (b) dendrite-like structure is marked with a color mask and occupied the area from 10 to 15% on the surface.

According to the results of AFM studies we have found that three of samples have a similar type of surface topography (Figure 3): S2, S3, and S4. On their surfaces, two types of morphological structure could be revealed, drop-like and dendrite-like. More often, these two structures are mixed with each other, but there are areas on the sample surface where one of these structures dominates (Figure 4). Dendrite-like structure is absent on S1 surface; moreover, drop-like structure is modified here: instead of rounded shapes, drops have jagged edges. It could be seen that drop-like structure on this sample is mostly concentrated in scratches, which supposedly is the result of surface polishing. This fact could be considered as a consequence of low binding of the drop-like structure to the surface in case of S1. The sample S2 differs from S4 by the proportion of drop- and dendrite-like structures. On the surface of S4 the dendrite-like structure occupies near 30% of total area of the surface and is practically equal the area occupied by the drop-like structure. On the surface of S2 the relative area of dendrite-like structure was twice bigger than the same value for drop-like structure (Figure 5). Another difference between S2 and S4 is a height of dendrite and drop-like structures. It looks like the case of S2 in which a dendrite-like structure lies below the dot-like structure, while in case of S4 both structures have approximately the same height (see, e.g., Figure 3). The surface topography of S3 differs essentially: we can see (Figure 6) the grain-like structure with average grain diameter about 0.5 *μ*m and with the roughness (root mean square of the surface height) of sample surface about 2 nm. The dendrite-like structure for this sample is more ramified; there are multiple breaks in dendrite branches. The value of projected area of the dendrite-like structure of S3 lies in the middle between the same values for S4 and S2.

**Figure 7:**
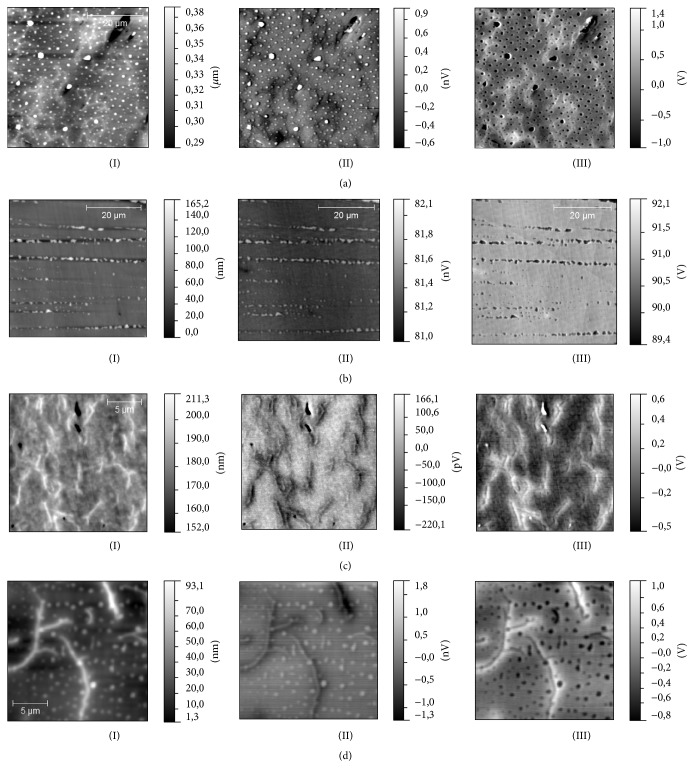
The results of MFM measurements: (I) topography of the samples, (II) MFM amplitude, and (III) MFM phase. (a) S2 sample, (b) S1 sample, (c) S3 sample, and (d) S4 sample.

**Figure 8:**
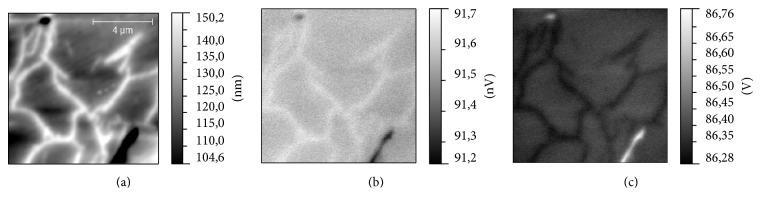
The results of EFM measurements of S2 sample: (a) topography of the sample, (b) EFM amplitude, and (c) EFM phase.

**Figure 9:**
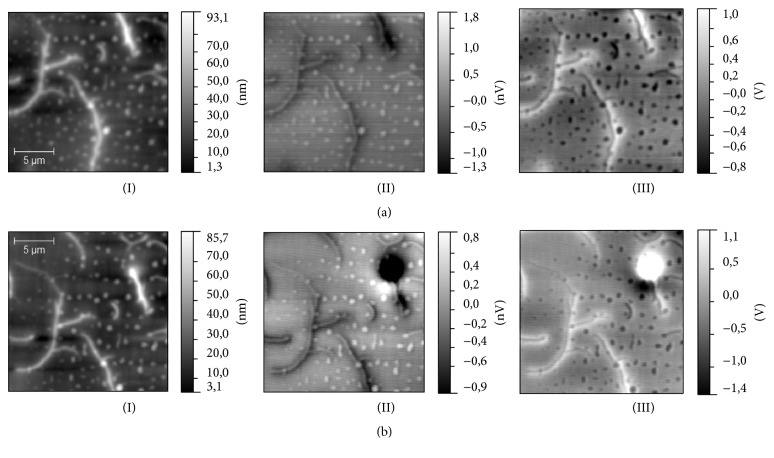
The results of MFM measurements in magnetic field: (I) topography of the sample, (II) MFM amplitude, and (III) MFM phase. (a) S4 sample in the absence of magnetic field; (b) S4 sample in the presence of magnetic field. 0.6 T was applied coaxial to *Y* direction and parallel to the surface.

The results of MFM measurements in zero magnetic field have revealed that both types (grain- and dendrite-like) of surface structures have the MFM contrast independently of a height on which a surface magnetization had been detected (Figure 7). These structures have been clearly observed even when the distance between the MFM probe and the surface was as large as 1 *μ*m; thus it could be concluded that the MFM contrast is not originated from the cross-talk with the topography. To check the possible nature of this contrast, the additional measurements have been performed. At first, two of these samples (S2 and S3) have been scanned in a noncontact electrostatic force microscopy (EFM) regime [30]. In this regime measurements were done with nonmagnetic probe; in our case it was PPP-NCHR probes (Nanosensors, Switzerland), while the scheme of measurements was similar to the measurements in MFM regime. It is known that the contrast in EFM images is proportional to the capacitive probe-sample electric force derivative and thus, in our case, could be evidence of nonuniform charge distribution on the surface. The results of measurements in EFM regime (Figure 8) have indicated that the contrast of both drop-like and dendrite-like structures is clearly observed on EFM amplitude and phase images. This inhomogeneous charge distribution could be responsible for high conductivity observed earlier in magnetic alkali borosilicate glasses [6]. This experimental fact brings up the question about the nature of MFM contrast observed in MFM measurements. Indeed the resemblance of the MFM and EFM contrast could be considered as the argument for an electrostatic nature of the MFM contrast. In MFM measurements a magnetic probe could be sensitive to the charge accumulated on the drop- or dendrite-like structures and instead of magnetic properties distribution reveals a surface potential distribution. To check this assumption, MFM measurements in external magnetic field have been carried out. Magnetic field in the range of ±0.7 T was applied parallel to the sample surface. Measurements have been performed with MFM-HC probes with hard magnetic coating. MFM contrast of both types of surface structures have not changed in magnetic field. Nevertheless, it has been found that in case of S1 and S3 samples additional disk-shaped structures were visualized in MFM images. Presumably, these disks are located in the subsurface layers, up to 10 *μ*m in sizes and oriented orthogonal to the external magnetic field (Figure 9). In contrast to drop- and dendrite-like structures (they become static in magnetic field and could be distinguished also in the EFM images) the disks have appeared only in external magnetic field. We suppose that we have not observed an alteration of magnetic contrast of drop-like and dendrite-like structures in the external magnetic field due to the fact that the value of this field was significantly lower than the coercive field of magnetic clusters (or these types of structures) in the matrix skeleton. The studies of magnetization [29] and field dependency of magnetostriction [7] argue for this assumption. Finally, we have tested the magnetic properties of S2, S3, and S4 samples after the one-step chemical etching by acid and only S3 had demonstrated explicit magnetic properties after this procedure. According to the preliminary results of chemical analysis of obtained porous glasses the most part of iron atoms in S2 and S4 samples had been removed at etching. So, according to the results of our measurements, we can conclude that technological regime used for production of S3 sample is more optimal for fabrication of porous magnetic alkali borosilicate glasses by induction melting.

**Figure 10:**
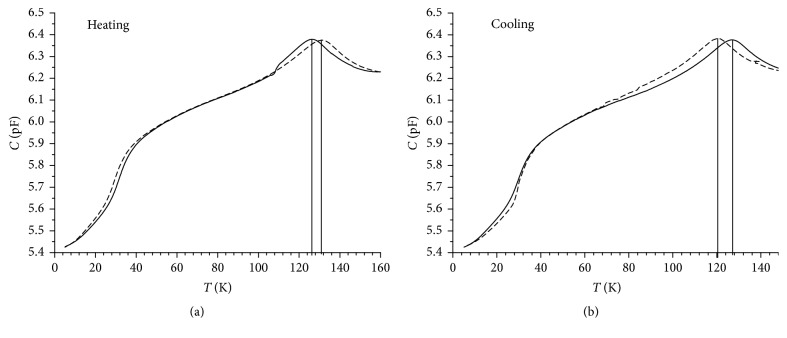
Temperature dependence of capacitance *C* for S3 + KDP nanocomposite: (a) on heating in zero field (solid line) and at* B* = 10 T (dash line); (b) on cooling in zero field (dash curve) and at* B* = 10 T (solid curve). Vertical lines indicate the positions of maximum on the *C*(*T*) curves.

On the last stage we have prepared the nanocomposites with embedded ferroelectric KH2PO4 (KDP) on the basis of macroporous (the average pore diameter was about 60 nm) S3 glasses. In Figure 10 the temperature dependence of capacities of these nanocomposites is presented: Figure 10(a) on heating at zero magnetic field and at* B* = 10 T; Figure 10(b) on cooling at the same conditions. The ferroelectric phase transition (PT) in massive KDP takes place at the Curie temperature of *T*_*C*_ ~ 122 K. This value is close to* T* = 126 K, at which the maximum of capacity *C*(*T*) is observed for S3+KDP nanocomposite. A growth of *T*_*C*_ with decreasing of KDP nanoparticles size has been observed previously [31] and it is a result of size effect. The position of the *C*(*T*) anomaly observed at* T* = 126 K in the present work corresponds well to the dependence of *T*_*C*_ versus the average pore size reported in this paper [31]. Application of magnetics field with* B* = 10 T leads to a shift in the position of *C*(*T*) maximum to ~132 K on heating. In the bulk KDP the ferroelectric PT belongs to the order-disorder type. The temperature hysteresis between maxima in the dielectric permittivity *ε*(*T*) observed on cooling and heating does not exceed 1 K. Compared to Figures 10(a) and 10(b) one can see that in nanocomposites S3+KDP this hysteresis is about 5 K and does not depend on the regime of field application. These results confirm a possibility of governing by the ferroelectric phase transition using magnetic fields. The physical mechanism of *T*_*C*_ shift is a presence of magnetostriction in this type of magnetic glasses [7, 32].

At measurements of magnetostriction the magnetic field up to* B* = 14 T had been applied in plane (in *X* and *Y* directions) and perpendicular (in *Z* direction) to the flat plate (4.8*∗*4.8*∗*0.54 mm^3^) of macroporous magnetic glasses at 4.2 ± 0.01 K. The magnetostriction coefficients in *XY* plane and in *Z* directions at 0 < *B* < 1 T do not differ from zero, but in the diapason 1–14 T they demonstrate the linear growth at field increasing. The volume coefficient Δ*V*/*V* of magnetostriction achieves 2.3 · 10^−5^ at* B* = 14 T. The values of linear coefficients in the *X* and *Y* directions are practically the same (8.8 · 10^−6^), but Δ*L*/*L* in the *Z* direction (5.6 · 10^−6^) is a little bit smaller. This fact can be explained by the peculiarities through porous glasses production. We have used the thin flat plates and the etching process has predominantly developed from the plate surfaces but not from the lateral ends.

## 4. Conclusion

SEM, AFM, and MFM studies of nonporous ferriferous alkali borosilicate glasses prepared by induction melting confirm the phase separation on a chemically unstable phase (CUP) and stable skeleton. CUP is presented by the dendrite-like structure of interconnected channels that is a necessary condition for porous glasses production. A skeleton consists of silica with iron-rich agglomerates. Using independent carbon markers it is shown that the uniform distribution of initial components is provided at melting due to convection and electromagnetic agitation. Using the obtained results we have optimized the melting regimes and thermal treatment of ferriferous glasses with magnetic properties. The dielectric studies of nanocomposites on the basis of macroporous glasses embedded into the pores ferroelectrics KH_2_PO_4_ have shown the possibility of affecting the ferroelectric phase transition by applied magnetic fields. These results open a possibility of producing the porous magnetic matrices and multifunctional nanocomposite materials with coexisting ferroelectric and magnetic orderings with developed interface based on these porous glasses.

## Figures and Tables

**Table 1:** Melting and phase separation parameters.

Sample	*T*_melt_, °C	*T*_sep_, °C	*T*_dur_, melting duration, min	*T*_ph_, time of heat treatment, hours
S1	1550 ± 30	—	30	—
S2	1580 ± 30	560 ± 2	30	120
S3	1520 ± 30	560 ± 2	90	120
S4	1480 ± 30	560 ± 2	60	120

**Table 2:** Element composition of two-phase magnetic glasses.

Sample	Composition (mass %)					
	C	O	Na	Fe	Si	Others
S1	9.43	47.91	4.73	3.54	34.38	0.01
S2	5.83	50.85	3.21	0.84	38.38	0.89
S3	12.40	44.08	3.78	13.27	26.45	0.02
S4	4.52	48.82	4.06	6.98	34.96	0.66

## Additional Points

*Headligthes*. (i) Two-phase magnetic alkali borosilicate glasses have been produced by induction melting. (ii) Co-existence of drop-like and dendrite-like morphological structures has been shown. (iii) Uniform agitation of initial components have been proved. (iv) The optimal melting parameters and heat treatment regimes have been determined. (v) The governing of ferroelectric phase transition by application of external magnetic field is shown.
